# *Aquilaria crassna* Extract Exerts Neuroprotective Effect against Benzo[a]pyrene-Induced Toxicity in Human SH-SY5Y Cells: An RNA-Seq-Based Transcriptome Analysis

**DOI:** 10.3390/nu16162727

**Published:** 2024-08-16

**Authors:** Nattaporn Pattarachotanant, Suporn Sukjamnong, Panthakarn Rangsinth, Kamonwan Chaikhong, Chanin Sillapachaiyaporn, George Pak-Heng Leung, Valerie W. Hu, Tewarit Sarachana, Siriporn Chuchawankul, Tewin Tencomnao, Anchalee Prasansuklab

**Affiliations:** 1 Center of Excellence on Natural Products for Neuroprotection and Anti-Ageing (Neur-Age Natura), Faculty of Allied Health Sciences, Chulalongkorn University, Bangkok 10330, Thailand; nat.ahs11@gmail.com (N.P.); kamonwan.chaikhong@gmail.com (K.C.); chanin.s@chula.ac.th (C.S.); 2 Department of Clinical Chemistry, Faculty of Allied Health Sciences, Chulalongkorn University, Bangkok 10330, Thailand; suporn.su@chula.ac.th (S.S.); tewarit.sa@chula.ac.th (T.S.); 3 Chulalongkorn Autism Research and Innovation Center of Excellence (Chula ACE), Department of Clinical Chemistry, Faculty of Allied Health Sciences, Chulalongkorn University, Bangkok 10330, Thailand; 4 Department of Pharmacology and Pharmacy, Li Ka Shing Faculty of Medicine, The University of Hong Kong, Hong Kong, China; ptkrs@hku.hk (P.R.); gphleung@hku.hk (G.P.-H.L.); 5 Department of Biochemistry and Molecular Medicine, The George Washington University School of Medicine and Health Sciences, The George Washington University, Washington, DC 20037, USA; valhu@gwu.edu; 6 Department of Transfusion Medicine and Clinical Microbiology, Faculty of Allied Health Sciences, Chulalongkorn University, Bangkok 10330, Thailand; siriporn.ch@chula.ac.th; 7 College of Public Health Sciences, Chulalongkorn University, Bangkok 10330, Thailand

**Keywords:** agarwood, RNA sequencing, molecular docking, polycyclic aromatic hydrocarbons, neurite outgrowth, neurotoxicity, signaling pathways

## Abstract

Benzo[a]pyrene (B[a]P) is known to inhibit neurodifferentiation and induce neurodegeneration. Agarwood or *Aquilaria crassna* (AC), a plant with health-promoting properties, may counteract the neurotoxic effects of B[a]P by promoting neuronal growth and survival. This study investigated the protective effect of AC leaf ethanolic extract (ACEE) on the B[a]P-induced impairment of neuronal differentiation. A transcriptomic analysis identified the canonical pathway, the biological network, and the differentially expressed genes (DEGs) that are changed in response to neuronal differentiation and neurogenesis. Several genes, including *CXCR4*, *ENPP2*, *GAP43*, *GFRA2*, *NELL2*, *NFASC*, *NSG2*, *NGB*, *BASP1*, and *NEUROD1*, in B[a]P-treated SH-SY5Y cells were up-regulated after treatment with ACEE. Notably, a Western blot analysis further confirmed that ACEE increased the protein levels of GAP43 and neuroglobin. B[a]P treatment led to decreased phosphorylation of Akt and increased phosphorylation of ERK in SH-SY5Y cells; however, ACEE was able to reverse these effects. Clionasterol and lupenone were identified in ACEE. Molecular docking showed that these two phytochemicals had significant interactions with CXCR4, GDNF family receptor alpha (GFRA), and retinoid X receptors (RXRs). In conclusion, ACEE may be a potential alternative medicine for the prevention of impaired neuronal differentiation and neurodegenerative diseases.

## 1. Introduction

Neurodegenerative diseases (NDDs) are chronic disorders with the primary characteristics of slow progressive damage and a loss of neurons, as well as an impairment in neuronal proliferation and differentiation in the central nervous system (CNS) [1,2]. This phenomenon leads to deficits in brain functions, including memory, communication, cognition, and movement. The most common NDDs are Alzheimer’s disease and Parkinson’s disease, both of which currently lack an efficient cure [3]. Protection is also difficult because the pathology is multifactorial [4,5]. Current therapies are primarily aimed at managing symptoms [2]. The development of preventative and therapeutic alternatives with high efficiency is needed. With the aim to impede neuron loss and preserve the number of viable neurons, the induction of neuronal proliferation and differentiation might be a potential therapeutic approach for NDDs [6,7].

While the causes of NDDs are diverse, several studies have established a link between these diseases and exposure to air pollutants, especially polycyclic aromatic hydrocarbons (PAHs) [8,9]. Benzo[a]pyrene (B[a]P), a highly studied PAH, is produced through incomplete combustion of fuels, as well as from the smoke of cigarettes and grilling foods [4]. B[a]P has multiple harmful effects on human health, including carcinogenesis, impairment of fertility, and neurotoxicity [10,11,12,13]. B[a]P causes impaired neurodifferentiation, induction of neuronal loss, deposition of plaque, cognitive decline [14,15,16,17,18], and suppression of synaptic vesicle exocytosis in hippocampal neurons [13]. Interestingly, B[a]P could induce severe behavioral deficits in mice. After B[a]P exposure, microglia and astroglia were activated and the expression of inducible nitric oxide synthase was up-regulated in the cortex and hippocampus of mice [17]. Nevertheless, the molecular mechanisms involved in B[a]P-induced neurotoxicity have not yet been fully understood.

*Aquilaria crassna* (AC), or agarwood, is commonly consumed as an ingredient in foods such as biscuits, herbal soups, and instant noodles [19,20]. In traditional medicine in Southeast Asian countries, it is used as a sedative and for the treatment of diarrhea and inflammation-related problems such as joint pain [19]. It has been shown in an earlier study that the ethanolic extract of whole-tree agarwood could reduce isoproterenol-induced myocardial ischemia in rats [21]. In addition, its essential oil possesses an anxiolytic and circadian regulatory effect [22,23]. The leaf and heartwood of AC also possess neuritogenic and neuroprotective constituents [24,25]. According to previous reports, the leaf extract of AC showed protective effects against high glucose-induced neurotoxicity and neurite outgrowth impairment in neuroblastoma cells via up-regulated expressions of teneurin-4 and growth-associated protein 43 (GAP43). Similarly, the leaf extract of AC alleviated high glucose-induced aging in *Caenorhabditis elegans* via the up-regulation of genes related to the DAF-16/FoxO pathway [26]. Moreover, we found that the leaf extract of AC normalized cell cycle progression in B[a]P-treated neuronal cells by modulating the aryl hydrocarbon receptor (AhR)/cytochrome P450 (CYP) 1A1/cyclin D1 pathway and enhanced xenobiotic detoxification and lifespan in *C. elegans* exposed to B[a]P [27]. Nevertheless, the ability of this extract on B[a]P-induced impaired neuronal differentiation has not been explored. The present study aimed to investigate the properties and mechanisms of AC leaf extract in restoring B[a]P-induced impairment of SH-SY5Y cell differentiation by transcriptome analysis using the RNA sequencing (RNA-Seq) technique, which was further validated using quantitative Reverse Transcription PCR (RT-qPCR) and Western blot analysis. The constituents in the extract, which may bind to the receptors related to neurodifferentiation, were also investigated using in silico molecular docking.

## 2. Materials and Methods

### 2.1. Chemicals and Reagents

B[a]P and ammonium persulfate were obtained from Sigma-Aldrich (St. Louis, MO, USA). Ethyl alcohol (ethanol) and dimethyl sulfoxide (DMSO) were from RCI Labscan (Bangkok, Thailand). Phenylmethyl sulphonyl fluoride was obtained from US Biological (Cleveland, OH, USA). Retinoic acid (RA) and the Immobilon Chemiluminescence horseradish peroxidase (HRP) substrate reagent were purchased from Merck (Darmstadt, Germany). The penicillin–streptomycin solution, fetal bovine serum (FBS), and Dulbecco’s Modified Eagle Medium (DMEM)/high glucose were from HyClone (Logan, UT, USA). MTT was purchased from BioBasic (Markham, ON, Canada). The GENEzol™ reagent was purchased from Geneaid Biotech Ltd. (New Taipei City, Taiwan). The acrylamide/bis-acrylamide solution was purchased from HiMedia (Maharashtra, India). The rabbit monoclonal antibodies including GAP43, phospho-Akt (Ser473), Akt, phospho-p38 MAPK (Thr180/Tyr182), p38 MAPK, phospho-Erk1/2 MAPK (Thr202/Tyr204), Erk1/2 MAPK, β-actin, and the anti-rabbit IgG HRP-conjugated antibodies were purchased from Cell Signaling Technology (Beverly, MA, USA). The mouse monoclonal neuroglobin antibody was from Santa Cruz Biotechnology (Dallas, TX, USA). The anti-mouse IgG HRP-linked antibody was obtained from Abcam (Cambridge, UK).

### 2.2. Plant Extract Preparation

AC leaves were obtained from the Her Royal Highness Princess Maha Chakri Sirindhorn Herb Garden, Thailand. The protocols for accessing and collecting plant material were conducted following relevant national guidelines, including obtaining the necessary permits and adhering to ethical academic standards throughout the process. Permission for the collection of plant material was given by the authorities at the herbal garden. The identification of plant species was carried out by Mrs. Parinyanoot Klinratana at the Professor Kasin Suvatabhandhu Herbarium (Chulalongkorn University, Thailand) and deposited with voucher specimen number A17634(BCU). The method of preparation of the AC leaf ethanolic extract (ACEE) has been described previously [27]. In brief, the fine powder of dried AC leaves was macerated in absolute ethanol at a ratio of 1:10 (*w*/*v*) under room temperature for three consecutive days. The resulting extract was filtered and concentrated under a vacuum in a rotary evaporator. A 100 mg/mL stock solution was prepared by dissolving the dried AC extract in DMSO and stored at −20 °C for further experimental uses.

### 2.3. Cell Culture

Human neuroblastoma SH-SY5Y cells were purchased from Cytion (CLS Cell Lines Service, Heidelberg, Germany). The cells were maintained and grown in DMEM/high glucose supplemented with 10% FBS and 1X penicillin–streptomycin solution at a 37 °C humidified atmosphere containing 5% CO_2_.

### 2.4. Drug Treatment

The differentiation in SH-SY5Y cells was induced by 10 µM RA in DMEM containing 1% FBS for 24 h [26]. Then, the differentiated cells were treated with either 5 or 25 µg/mL ACEE in combination with B[a]P at varied concentrations (1, 5, and 10 µM) for an additional 48 h. These two concentrations of ACEE were chosen based on our previous studies [27].

### 2.5. MTT Viability Assay

The viability of SH-SY5Y cells were evaluated by adding the MTT reagent (5 mg/mL) and incubated for four hours at 37 °C. After that, the solution was removed, and DMSO was subsequently added to the well to solubilize the purple formazan crystal products. Finally, the absorbance was read at 550 nm using a microplate reader (PerkinElmer, Hopkinton, MA, USA).

### 2.6. Neurite Outgrowth Assay

To study the neurite outgrowth formation in SH-SY5Y cells, the quantification of neurite-bearing cells and neurite length was conducted using the protocol described in our previous report [26]. The cells were imaged with a CKX53 culture microscope (Olympus Corporation, Tokyo, Japan). The cell was considered neurite-bearing when it had at least one neurite longer than the diameter of the cell body. Neurite length was measured as the length of the longest neurite per neuron using the Motic Images Plus 3.0 software (Motic Hong Kong Ltd., Kowloon, Hong Kong). Lastly, the neurite-bearing cells and neurite length were analyzed from at least ten random microscopic fields and calculated as the percentage of RA-treated cells.

### 2.7. Analysis of Transcriptome Profiling

Total RNA was prepared using the GENEzol™ reagent (Geneaid) following the manufacturer’s protocol. Then, the Nanodrop™ 2000 spectrophotometer (Thermo Fisher Scientific, Waltham, MA, USA) was used for the determination of RNA concentration and purity. The RNA samples with an acceptable purity A260/A280 ratio of 2.0 and A260/A230 ratio in the range of 2.0–2.2 [28] were submitted to BGI Genomics (Hong Kong) using the DNBSEQ™ sequencing technology platform for transcriptome RNA sequencing (RNA-Seq). The comparison of transcriptome profiles was performed using a Poisson distribution to identify differentially expressed genes (DEGs) between treatment groups, by considering the genes with a *p*  <  0.05 and FDR  <  0.05. Ingenuity Pathway Analysis (IPA) software version number 84978992 (Qiagen Inc., Santa Clarita, CA, USA) was also employed to investigate the potential pathways, biological functions, diseases, and regulatory networks associated with the identified DEGs. The Fisher’s exact test was calculated in IPA for an analysis of the relevant pathways and functional categories, where *p* < 0.05 was considered significant.

### 2.8. RT-qPCR Analysis

RT-qPCR was performed to analyze gene expression and validate the results of transcriptomic sequencing. One µg of each RNA sample, extracted from the cells using the GENEzol™ reagent (Geneaid), was reverse-transcribed into complementary DNA (cDNA) using the Maxime™ RT PreMix kit containing the oligo dT15 primer (iNtRON Biotechnology, Gyeonggi, Republic of Korea). The qPCR amplification of the cDNA template was carried out in a CFX 96 Real-Time PCR Detection System (Bio-Rad, Hercules, CA, USA) using the iTaq Universal SYBR Green Supermix (Bio-Rad) and the specific primers for target genes listed in Table 1. The gene expression levels were calculated relative to the expression of the reference ACTB (β-actin) gene using the 2^−ΔΔCT^ method.

### 2.9. Western Blot Analysis

An analysis of protein expression by Western blotting was performed to confirm the selected gene expression changes and identify the molecular signaling pathways associated with the effects of B[a]P and/or ACEE in SH-SY5Y cells. An NP-40 lysis buffer was used to extract the total protein from the SH-SY5Y cells. The concentration of protein samples was determined using the Bradford reagent (Bio-Rad). After gel electrophoresis, the separated proteins were transferred onto a polyvinylidene fluoride membrane. Following the transfer, the membrane was blocked and incubated overnight with primary antibodies, including GAP43 (1:5000), *p*-p38 (1:2000), p38 (1:5000), *p*-ERK (1:2000), ERK (1:5000), *p*-Akt (1:5000), Akt (1:5000), neuroglobin (1:2000), and β-actin (1:5000) as the normalization control. Finally, the protein band of interest was visualized through incubation with the HRP-conjugated secondary antibody, followed by a chemiluminescence detection reagent. Quantification of the band intensity was conducted using ImageJ software version 1.53 (NIH, Bethesda MD, USA).

### 2.10. Molecular Docking

The X-ray crystallographic structural data of CXCR4 (PDB ID: 3ODU) [29], GFRA1 (PDB ID: 4UX8) [30], GFRA2 (PDB ID: 5MR4) [31], RXRα (PDB ID: 1FM9) [32], RXRβ (PDB ID: 1UHL) [33], and RXRγ (PDB ID: 2GL8) [34] were retrieved from the Protein Data Bank (RCSB PDB; www.rcsb.org, accessed 5 January 2024). The structures of the phytochemical compounds used in this study were gained from the PubChem database. The preparation of ligands and proteins was described in our previous study [26,35]. The docking analysis was conducted using AutodockTools 1.5.6 (The Scripps Research Institute, San Diego, CA, USA), employing the Lamarckian Genetic Algorithm with default parameters.

### 2.11. Data Analysis

The data were expressed as the mean ± standard deviation (SD) from at least three independent experiments. To analyze the difference of mean values among more than two groups, one-way ANOVA was employed, followed by a post hoc Tukey’s test for pairwise comparison, where statistical significance was considered at the *p* < 0.05 level.

## 3. Results

### 3.1. ACEE Promoted Neuronal Differentiation of B[a]P-Treated SH-SY5Y Cells

**Figure 1:**
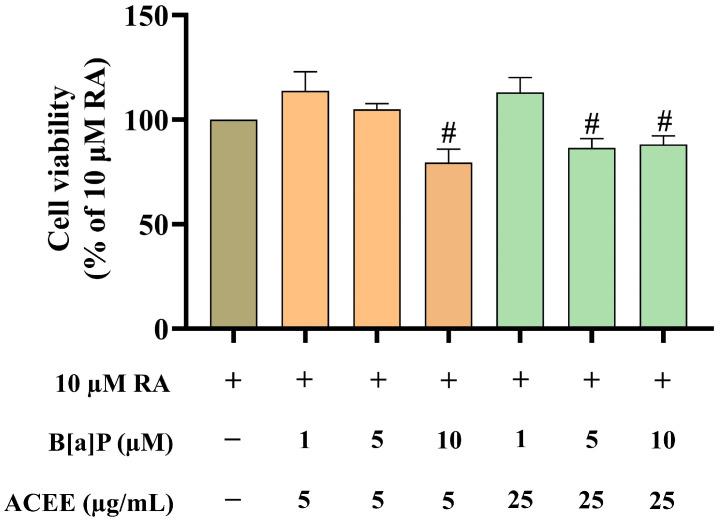
The cytotoxicity of B[a]P plus ACEE at varied concentrations in RA-differentiated SH-SY5Y cells was evaluated by MTT viability assay. Data are represented as the mean ± SD of the percentage of cell viability relative to the RA-alone-treated group. # *p* < 0.05 vs. RA-alone-treated group.

To select the appropriate concentrations of ACEE and B[a]P for the study of neuronal differentiation, we evaluated the toxicity of ACEE plus B[a]P at varied concentrations in RA-induced differentiated SH-SY5Y cells. The MTT viability results (Figure 1) showed that 10 µM B[a]P in combination with 5 µg/mL ACEE significantly reduced the cell viability to 79.6%. When 5 and 10 µM B[a]P were used in combination with 25 µg/mL ACEE, the cell viability was reduced to 86.5% and 88.1%, respectively. In contrast, when 1 µM B[a]P was used, no cell toxicity resulted when either 5 or 25 µg/mL ACEE was used. Therefore, combinations of 1 µM B[a]P and ACEE (5 and 25 µg/mL) were selected for subsequent experiments.

**Figure 2:**
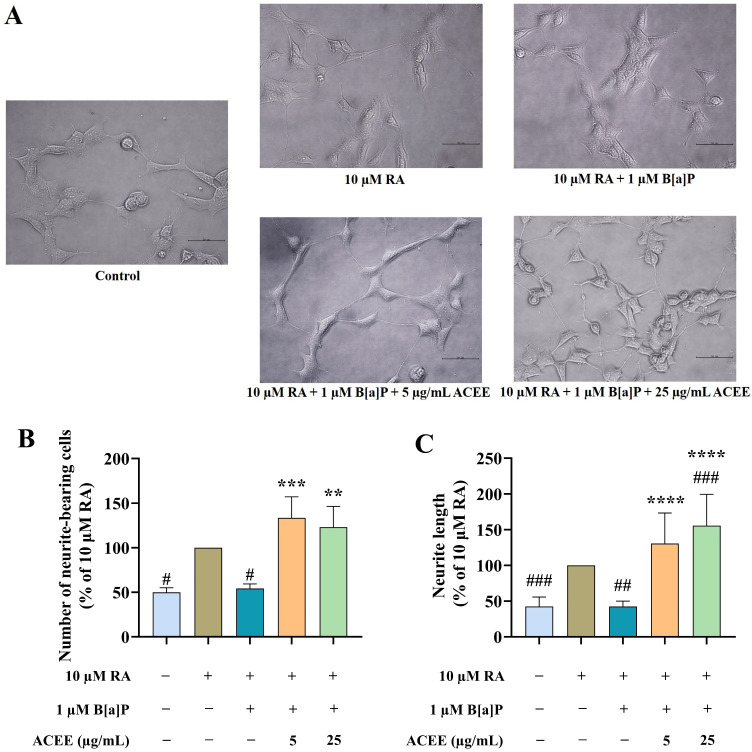
ACEE promoted neuronal differentiation in B[a]P-treated RA-differentiated SH-SY5Y cells. (**A**) The images of the neurite outgrowth process were captured using a microscope at 40× magnification (scale bar: 50 µm). (**B**) The percentage of neurite-bearing cells, and (**C**) the percentage of neurite length in SH-SY5Y cells after treatments. Data are presented as the mean ± SD of at least three independent experiments. # *p* < 0.05, ## *p* < 0.01, ### *p* < 0.0005 vs. RA-treated group; ** *p* < 0.01, *** *p* < 0.0005, **** *p* < 0.0001 vs. RA + B[a]P-treated group.

Next, the effects of B[a]P and/or ACEE on neurite outgrowth, a major phenotype of differentiated neurons, were investigated. The differentiation was successfully developed in RA-treated SH-SY5Y cells, which showed an increased percentage of neurite-bearing cells and neurite length (Figure 2). B[a]P at 1 µM significantly inhibited the formation of neurite-bearing and neurite elongation compared to RA-treated cells by 50% (Figure 2B,C). However, the co-treatments of ACEE at 5 and 25 µg/mL restored those impairments in differentiated neurons caused by B[a]P.

### 3.2. Transcriptome Profiling of SH-SY5Y Cells among Treatment Conditions

#### 3.2.1. The Quality of RNA Sequencing Data

To further investigate how the B[a]P and ACEE treatments altered the transcriptome profiles of RA-differentiated SH-SY5Y cells, RNA-Seq analyses were conducted. After filtering the poor-quality reads, the remaining 44,651,230, 44,043,028, and 45,183,328 high-quality clean reads were retained for the RA (control), RA + B[a]P, and RA + B[a]P + ACEE samples, respectively. In summary, from the RNA-Seq data, approximately 92.72% of the total clean reads were uniquely aligned and mapped to the reference genome across all samples.

#### 3.2.2. DEGs under Different Treatment Conditions

We identified 4189 genes as significantly differentially expressed in B[a]P-treated cells compared to the control (RA) and 4113 genes as significantly differentially expressed in cells co-treated with B[a]P and ACEE relative to the B[a]P-treated cells (*p* < 0.05 and FDR < 0.05; Table 2). The full lists of DEGs between treatment groups are presented in Supplementary Tables S1 and S2.

#### 3.2.3. Biological Pathways and Network Analyses of DEGs

The DEGs among the different treatment conditions in differentiated SH-SY5Y cells were analyzed to predict the DEG-associated diseases/disorders, biological functions, and canonical pathways by using IPA software. “Neurological disease” was among the top diseases/disorders analyzed from both DEGs of the cells treated with B[a]P relative to the control (3014 genes; *p* = 3.40 × 10^−18^ – 8.18 × 10^−106^; Table 3) and DEGs of the cells co-treated with B[a]P and ACEE relative to the B[a]P-treated cells (2969 genes; *p* = 3.84 × 10^−17^–2.26 × 10^−116^; Table 3). The identified DEGs induced by B[a]P treatment were shown to be significantly linked to neurological diseases, including “brain tumor”, “motor dysfunction or movement disorder”, “neuromuscular disease”, “cognitive impairment”, and “progressive neurological disorder” (*p* < 0.05; Table 4). Several processes related to nervous system development and functions, including “neuritogenesis”, “development of neurons”, “morphology of the nervous system”, “proliferation of neuronal cells”, and “dendritic growth/branching”, were also significantly associated with DEGs (*p* < 0.05; Table 4).

In addition, DEGs of the cells co-treated with B[a]P and ACEE were associated with many types of neurological diseases, including “brain tumor”, “cognitive impairment”, “neuromuscular disease”, “dementia”, and “tauopathy” (*p* < 0.05; Table 4). The results also revealed that DEGs of the cells co-treated with B[a]P and ACEE were significantly associated with “neuritogenesis”, “development of neurons”, “morphology of the nervous system”, “proliferation of neuronal cells”, and “dendritic growth/branching” (*p* < 0.05; Table 4).

**Figure 3:**
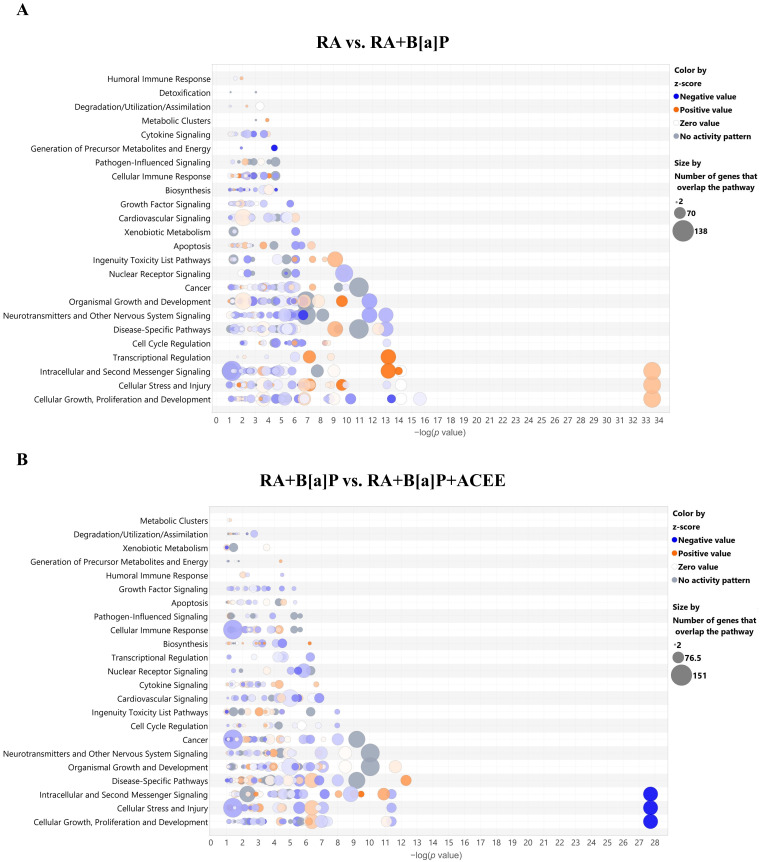
Analyses of the enriched canonical pathways associated with the identified DEGs among the treatment conditions in the RA-differentiated SH-SY5Y cells. The canonical pathways associated with DEGs were identified from (**A**) the cells treated with B[a]P and (**B**) the cells co-treated with B[a]P and ACEE relative to the control (RA). The DEG-associated canonical pathways were analyzed by IPA software using the list of DEGs from the RNA-Seq analysis. The bubble plot represents the predicted canonical pathways, with bubble size indicating the number of genes enriched in each pathway and bubble color representing the z-score. The orange bubble denotes a positive z-score and predicted activation state; the blue bubble denotes a negative z-score and predicted inhibition state.

The analysis of canonical pathways (Figure 3A) demonstrated that the identified DEGs from the cells induced by B[a]P treatment were significantly linked to alterations in the “Neurotransmitters and Other Nervous System Signaling” canonical pathway category, which includes the activation states of “Netrin Signaling” and “Semaphorin Neuronal Repulsive Signaling Pathway”, and the inhibition states of “Synaptogenesis Signaling Pathway”, “Ephrin Receptor Signaling”, “Neuropathic Pain Signaling in Dorsal Horn Neurons”, and “Oxytocin in Brain Signaling Pathway”. Moreover, the identified DEGs from the cells induced by B[a]P treatment were significantly linked to changes in the “Cellular Stress and Injury” canonical pathway category, such as the activation states of “EIF2 Signaling”, “Autophagy”, “Endoplasmic Reticulum Stress Pathway”, and “Necroptosis Signaling Pathway”.

Similarly, the significant associated changes in the “Neurotransmitters and Other Nervous System Signaling” canonical pathway category was observed in the results from DEGs of the cells when co-treated with B[a]P and ACEE (Figure 3B). Those changes include the activation states of “Reelin Signaling in Neurons”, “Ephrin Receptor Signaling”, “Semaphorin Neuronal Repulsive Signaling Pathway”, and “Glutamate Receptor Signaling” and the inhibition states of “Netrin Signaling”, “Neuropathic Pain Signaling in Dorsal Horn Neurons”, “Oxytocin in Brain Signaling Pathway”, “Synaptic Long-term Potentiation”, and “Synaptic Long-term Depression”.

**Figure 4:**
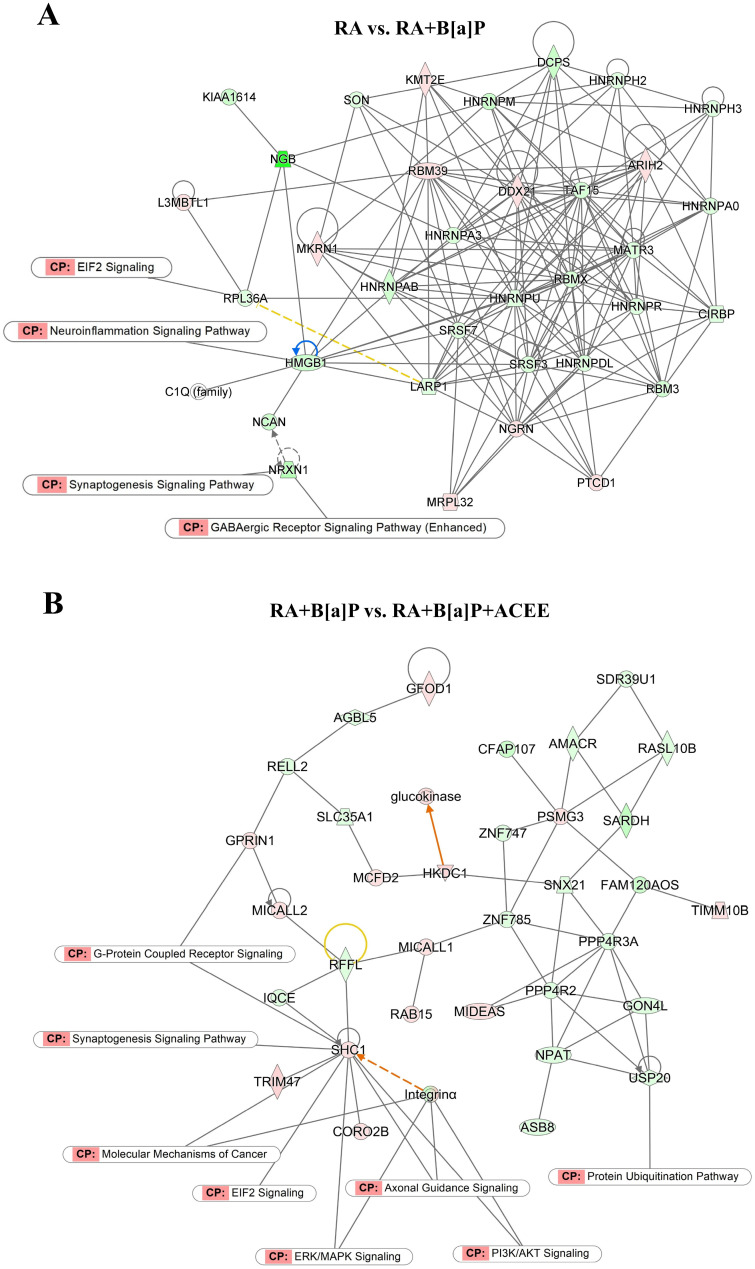
Network analyses of the identified DEGs among the different treatment conditions in theRA-differentiated SH-SY5Y cells. Graphical representation of biological networks obtained from the identified DEGs of (**A**) the cells treated with B[a]P and (**B**) the cells co-treated with B[a]P and ACEE relative to the control (RA). Red indicates up-regulation, while green indicates down-regulation. Orange line indicates predicted stimulation, blue line indicates predicted inhibition, yellow line indicates inconsistent findings, and grey line indicates effect not predicted. Solid uninterrupted line represents a direct action and dashed interrupted line represents an indirect action.

Biological networks of the identified DEGs among the different treatment groups were also assessed using IPA software. The analysis revealed a representative interactome network that illustrates the connections of DEGs from the control (RA) and B[a]P-treated cells in multiple signaling pathways, including “EIF2 Signaling”, “Neuroinflammation Signaling Pathway”, “Synaptogenesis Signaling Pathway”, and “GABAergic Receptor Signaling Pathway” (Figure 4A). Interestingly, some canonical pathways, including “EIF2 Signaling” and “Synaptogenesis Signaling Pathway”, were also significantly associated with DEGs from the cells co-treated with B[a]P and ACEE (Figure 4B).

### 3.3. RT-qPCR Validation of Selected DEGs

**Figure 5:**
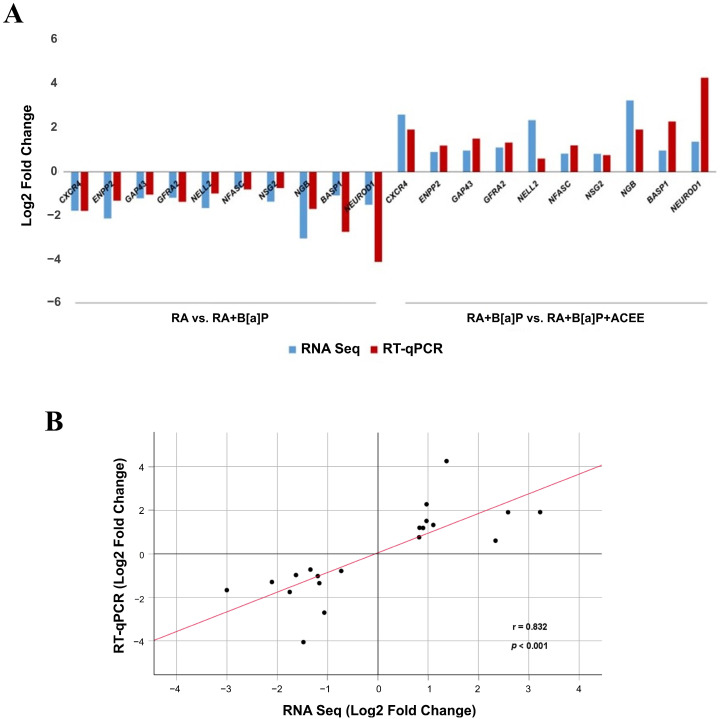
Correlation analysis of gene expression levels between the transcriptome (RNA-Seq) data and RT-qPCR data. (**A**) The relative log2 fold change values obtained from RNA-Seq (blue) and RT-qPCR (red). (**B**) The correlation plot comparing the log2 fold change values from RNA-Seq data (X-axis) and RT-qPCR data (Y-axis). Each point represents the intersection of two expression values from a pair of treatment groups: RA vs. RA + B[a]P and RA + B[a]P vs. RA + B[a]P + ACEE, respectively. Regression line and Spearman’s correlation coefficient are shown.

To further confirm the reliability of the transcriptome (RNA-Seq) data, the expression levels of ten selected DEGs (*CXCR4*, *ENPP2*, *GAP43*, *GFRA2*, *NELL2*, *NFASC*, *NSG2*, *NGB*, *BASP1*, and *NEUROD1*) were additionally examined by RT-qPCR. Details of all selected DEGs and their reported changes related to neuronal differentiation are provided in Table 5. The RNA-Seq analysis and RT-qPCR results yielded similar gene expression profiles (Figure 5A). A strong correlation was found between the log2 fold changes from both methods, with a Spearman’s correlation coefficient of 0.832 (*p* < 0.001) (Figure 5B).

**Figure 6:**
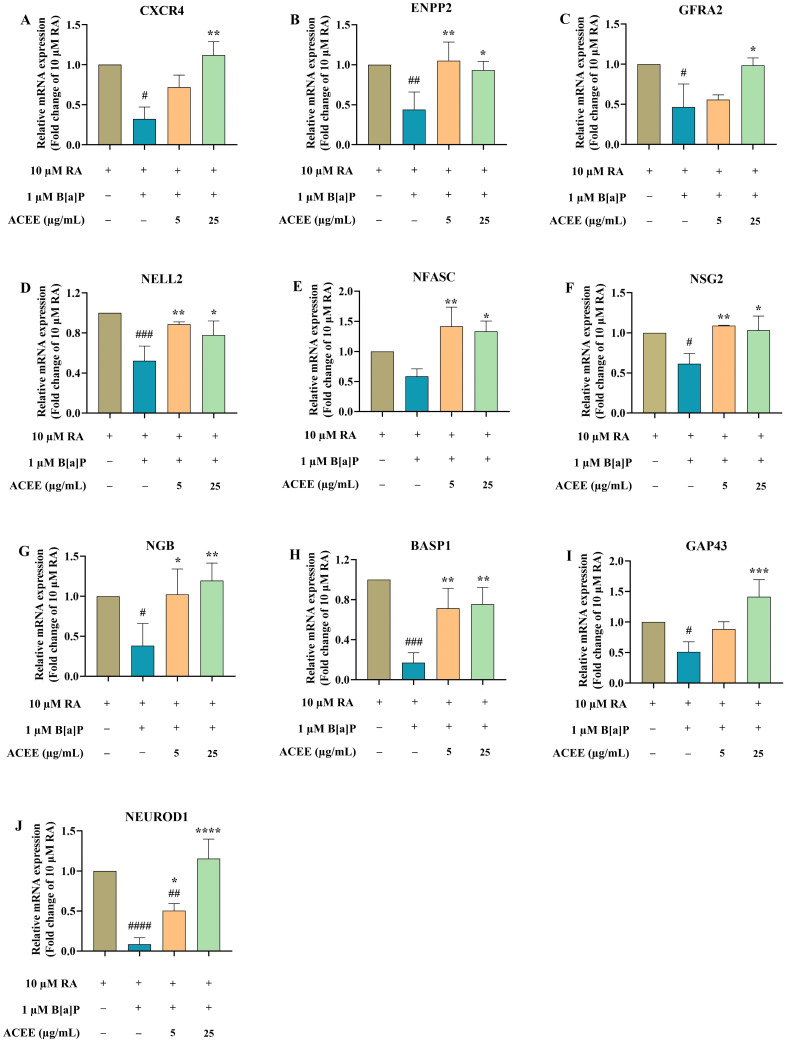
The effects of B[a]P and ACEE on the mRNA expressions of ten selected DEGs in RA-differentiated SH-SY5Y cells determined by RT-qPCR. The relative gene expression levels of (**A**) *CXCR4*, (**B**) *ENPP2*, (**C**) *GAP43*, (**D**) *GFRA2*, (**E**) *NELL2*, (**F**) *NFASC*, (**G**) *NSG2*, (**H**) *NGB*, (**I**) *BASP1*, and (**J**) *NEUROD1* normalized against ATCB (β-actin) expression. All data are presented as the mean ± SD of at least three independent experiments. # *p* < 0.05, ## *p* < 0.01, ### *p* < 0.0005, #### *p* < 0.0001 vs. RA-treated group; * *p* < 0.05, ** *p* < 0.01, *** *p* < 0.0005, **** *p* < 0.0001 vs. RA + B[a]P-treated group.

The mRNA expression levels of ten selected DEGs were analyzed by RT-qPCR (Figure 6). Compared to the control (RA-differentiated cells), treatment with B[a]P at 1 µM caused changes in the mRNA expressions of all selected DEGs except *NFASC* (Figure 6E). The levels of gene expression of *CXCR4*, *ENPP2*, *GFRA2*, *NELL2*, *NSG2*, *NGB*, *BASP1*, *GAP43*, and *NEUROD1* were significantly decreased by B[a]P treatment (*p* < 0.05), ranging from 38.5% (for *NEUROD1*) to 91.3% (for *NSG2*) (Figure 6A–D,F–J). However, these changes were substantially restored to normal levels by ACEE treatment, particularly at 25 µg/mL, suggesting the involvement of these genes in B[a]P-induced neurotoxicity and the neuroprotective mechanisms of ACEE.

### 3.4. Neuroprotective Effect of ACEE Is Mediated through Neuroglobin Up-Regulation via Akt- and ERK-Dependent Signaling Pathways

**Figure 7:**
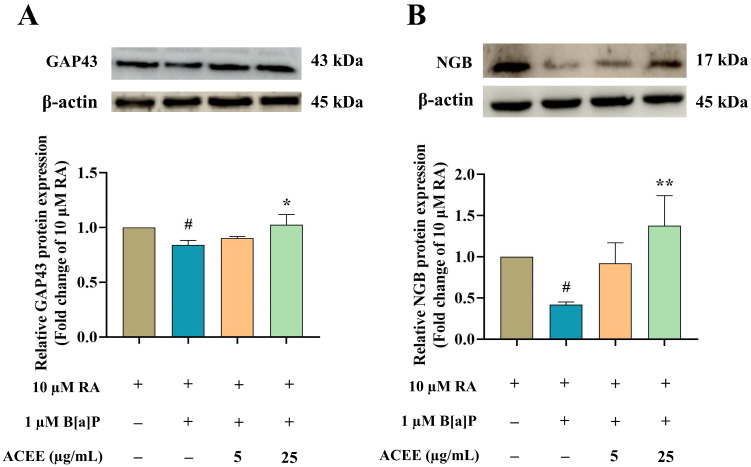
ACEE stimulated protein expression of GAP43 and NGB in B[a]P-exposed RA-differentiated SH-SY5Y cells. Representative Western blot images and bar graph representing (**A**) the relative GAP43 expression level and (**B**) the relative NGB expression level normalized against β-actin. All data were presented as the mean ± SD of at least three independent experiments. # *p* < 0.05 vs. RA-treated group; * *p* < 0.05, ** *p* < 0.01 vs. RA + B[a]P-treated group.

To elucidate the mechanisms underlying the B[a]P neurotoxicity and neuroprotective mechanisms of ACEE, the changes in transcriptional expression of two identified DEGs, namely, growth-associated protein 43 (GAP43) and neuroglobin (NGB), were studied using Western blot analysis. GAP43 is a common marker of differentiating neurons. NGB is a neuron-specific hemoprotein of the globin family that helps promote neuronal survival under hypoxia and various stress conditions. Both GAP43 and NGB also play a critical role in neurite formation [50,51,52,53]. This study demonstrated that B[a]P treatment suppressed the expression of GAP43 and NGB in differentiated SH-SY5Y cells at both the transcriptional (Figure 6G,I) and protein (Figure 7A,B) levels. These alterations could be fully restored to normal when the cells were co-treated with 25 µg/mL ACEE (Figure 7).

**Figure 8:**
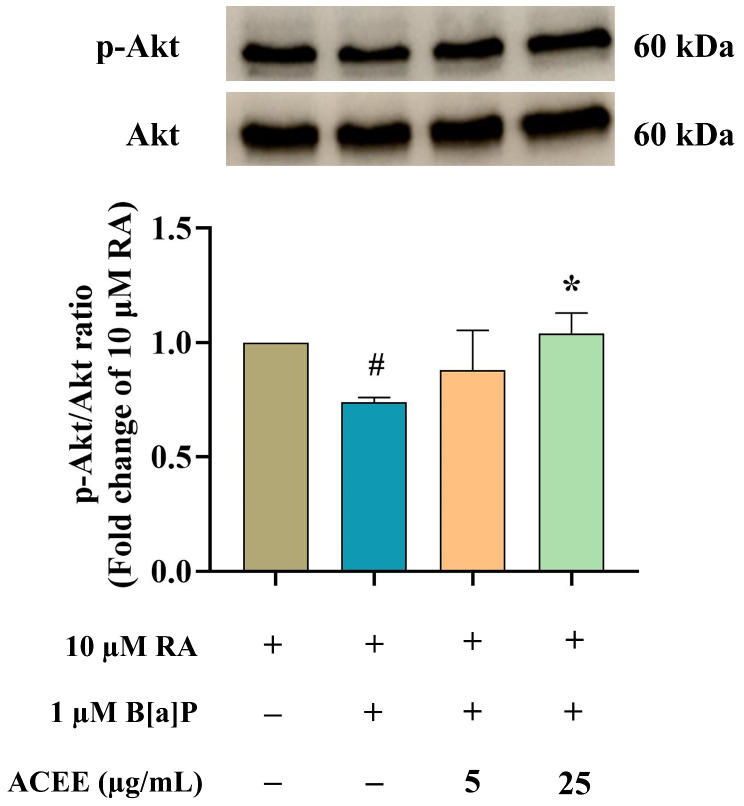
The effects of B[a]P and ACEE on the expressions of the Akt-dependent pathway in RA-differentiated SH-SY5Y cells determined by Western blot analysis. Representative Western blot images and bar graph representing the relative expression values of phosphorylated Akt (*p*-Akt) to total Akt. All data were presented as the mean ± SD of at least three independent experiments. # *p* < 0.05 vs. RA-treated group; * *p* < 0.05 vs. RA + B[a]P-treated group.

**Figure 9:**
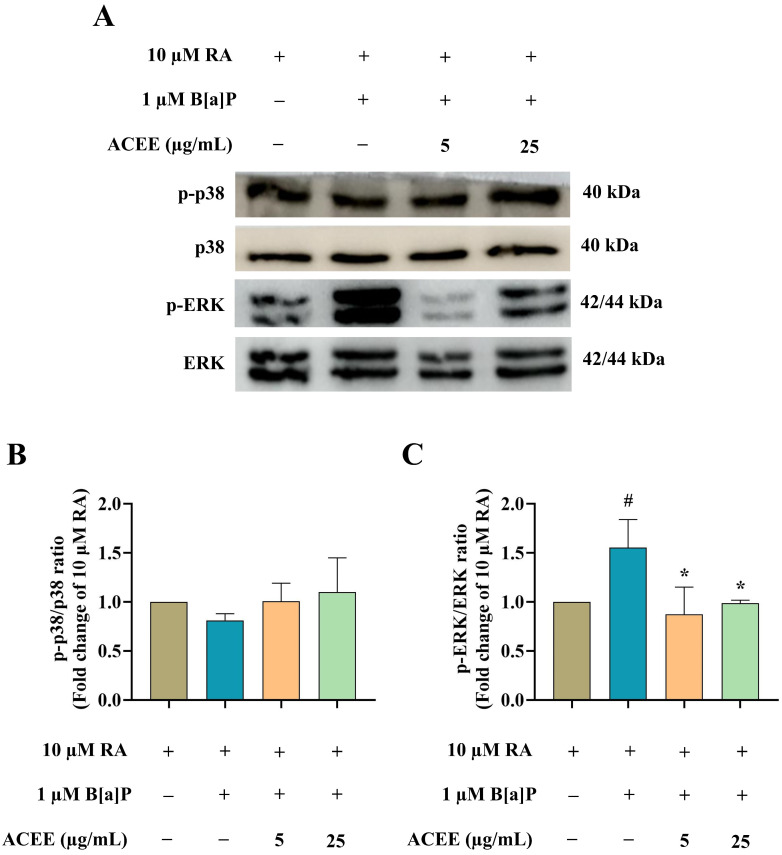
The effects of B[a]P and ACEE on the expressions of p38- and ERK-dependent pathways in RA-differentiated SH-SY5Y cells determined by Western blot analysis. (**A**) Representative Western blot images and bar graph representing (**B**) the relative expression values of phosphorylated p38 (*p*-p38) to total p38 and (**C**) the relative expression of phosphorylated ERK (*p*-ERK) to total ERK. All data are presented as the mean ± SD of at least three independent experiments. # *p* < 0.05 vs. RA-treated group; * *p* < 0.05 vs. RA + B[a]P-treated group.

The signaling pathways underlying the toxic effect of B[a]P and the protective mechanism of ACEE were further analyzed. The expressions of protein kinase B (Akt), p38, and extracellular regulated kinase (ERK) were investigated using Western blot analysis (Figure 8 and Figure 9). There was a significant decrease in the ratio of phosphorylated Akt (*p*-Akt) to total Akt (Figure 8) in RA-differentiated cells treated with 1 µM B[a]P, suggesting a decreased activation of the Akt-dependent pathway after exposure to B[a]P. Moreover, B[a]P treatment increased the ratio of phosphorylated ERK (*p*-ERK) to total ERK (Figure 9C), but no significant change was seen in the ratio of phosphorylated p38 (*p*-p38) to total p38 (Figure 9B), suggesting the activation of the ERK-dependent pathway. ACEE co-treatment restored the levels of *p*-Akt/Akt and *p*-ERK/ERK in B[a]P-induced differentiated neuronal cells (Figure 9).

### 3.5. Molecular Interaction among ACEE-Derived Phytochemical Constituents and CXCR4, GDNF Family Receptor Alpha (GFRA), and Retinoid X Receptor (RXR)

**Figure 10:**
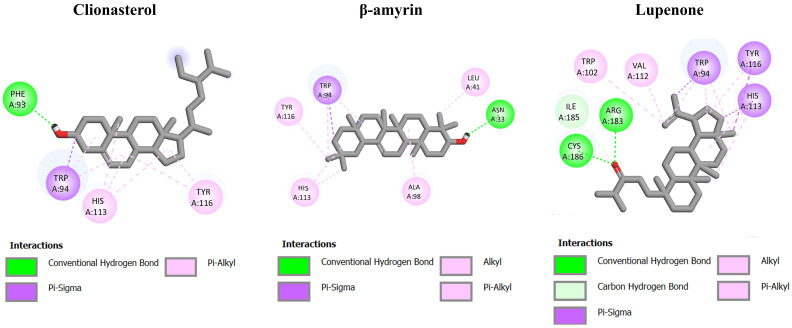
Molecular docking analysis of selected ACEE phytochemicals and CXCR4 receptor. The 2D interaction diagrams showing the docking poses of ACEE phytochemicals with the amino acids in CXCR4 binding site.

To further identify the interaction of ACEE on receptors of CXCR4, GFRA, and RXR, in silico molecular docking was performed on the ACEE-derived phytochemical compounds reported in our previous study [27] against those receptors, in comparison to NUCC-390, a CXCR4 agonist [54]; BT13, a GFRA agonist [55]; and 9-cis-retinoic acid, a RXR agonist [56]. As shown in Table 6, the binding energy of NUCC-390 was −8.11 kcal/mol for CXCR4. Based on the docking results in Table 6 and Figure 10, the three best-ranked phytochemicals, namely, clionasterol (−9.20 kcal/mol), β-amyrin (−8.69 kcal/mol), and lupenone (−8.69 kcal/mol) functioned as outstanding ligands with lower energy binding compared to the others and the standard agonist (NUCC-390). The binding energy of all phytochemical constituents is shown in Supplementary Tables S3–S8.

**Figure 11:**
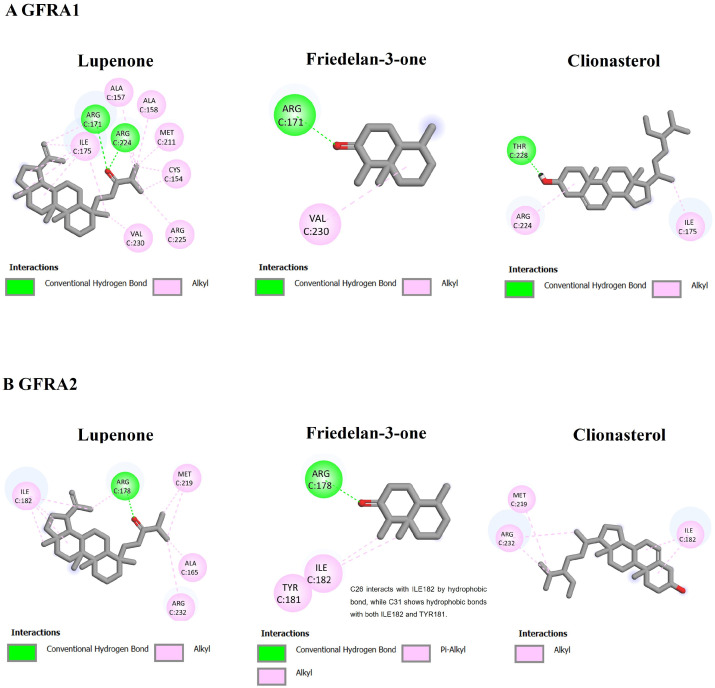
Molecular docking analysis of selected ACEE phytochemicals and GFRA receptors. The 2D interaction diagrams showing the docking poses of ACEE phytochemicals with the amino acids in (**A**) GFRA1 and (**B**) GFRA2 binding sites.

As shown in Table 6 and Figure 11, the binding energy of BT13, aGFRA agonist, was −5.31 and −5.55 kcal/mol for GFRA1 and GFRA2, respectively. The other three best-ranked compounds, lupenone (−7.66 kcal/mol), friedelan-3-one (−5.75 kcal/mol), and clionasterol (−5.43 kcal/mol), showed significant interaction with GFRA1 with the lower binding energy. Likewise, these best-ranked compounds exerted higher binding energy on GFRA2 when compared to the standard agonist, BT13. The binding energy was −6.86 kcal/mol for lupenone, −6.52 kcal/mol for friedelan-3-one, and −6.15 kcal/mol for clionasterol.

**Figure 12:**
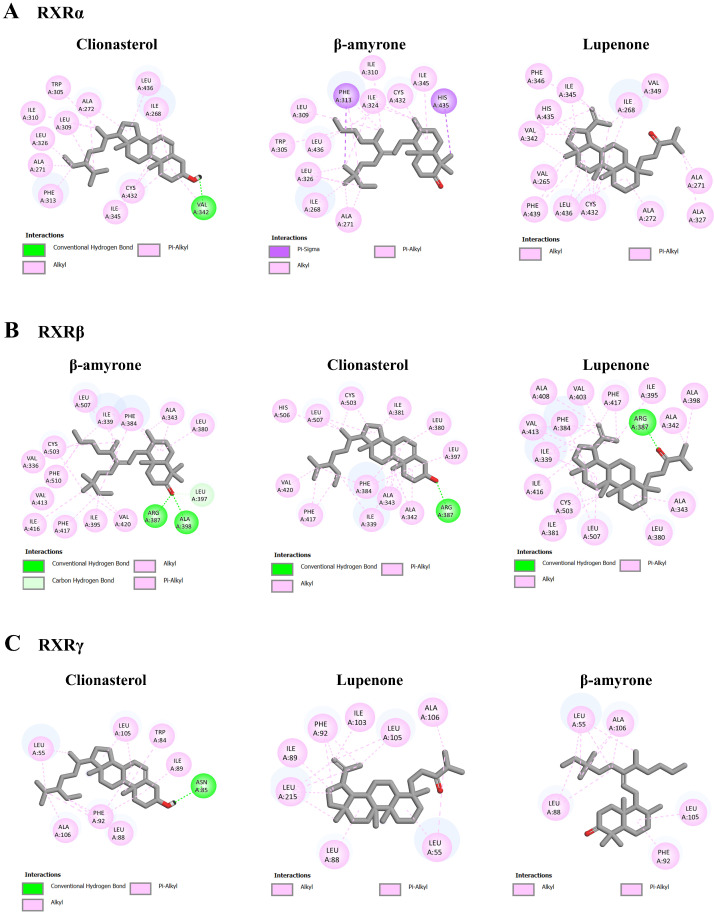
Molecular docking analysis of selected ACEE phytochemicals and RXR receptors. The 2D interaction diagrams showing the docking poses of ACEE phytochemicals with the amino acids in (**A**) RXRα, (**B**) RXRβ and (**C**) RXRγ binding sites.

As shown in Table 6 and Figure 12, the binding energy of 9-cis-retinoic acid, a RXR agonist, on RXRα, RXRβ, and RXRγ was −11.46, −10.63, and −7.79 kcal/mol, respectively. Based on the docking results, the three most potent ligands were clionasterol, β-amyrone, and lupenone, which exhibited lower binding energy than the standard agonist, 9-cis-retinoic acid. Together, clionasterol and lupenone could function as ligand agonists for all the target receptors with lower energy binding compared to the other compound in ACEE and the standard agonists.

## 4. Discussion

Benzo[a]pyrene (B[a]P), a typical PAH, is regarded as a pollutant in the environment and industry. People are exposed to B[a]P by consuming contaminated food or water, as well as inhaling cigarette smoke. B[a]P can pass through the blood–brain barrier and directly affect the CNS [57,58,59]. The bioactivity of B[a]P is linked to oxidative stress generation, interaction with the AhR, epigenetic changes, and immunosuppression. Intracellular reactive oxygen species generated during B[a]P metabolism through CYP enzymes results in the induction of oxidative stress in the CNS [60]. In zebrafish, chronic exposure to low levels of B[a]P has been shown to cause NDD-like features via the decreased ratio of brain weight to body weight and the loss of dopaminergic neurons, as well as the impairment of locomotor and cognitive activity [61]. Moreover, B[a]P is known to be teratogenic and carcinogenic by activating xenobiotic-metabolizing enzymes, such as CYP1A1 [27,62]. In addition, B[a]P at low concentrations suppresses neurodifferentiation in SH-SY5Y cells by inhibiting transglutaminase (TGase) expression and AhR signaling. B[a]P also reduced two neurodifferentiation markers, tyrosine hydroxylase and choline acetyltransferase, in PC12 cells [14,18]. Moreover, exposure to B[a]P during fetal development and childhood has a negative impact on neurodifferentiation and neurobehavioral development [63].

The SH-SY5Y cell line is widely used to study NDDs. The cells are derived from human neuroblastoma, making them more relevant for human disease studies compared to non-human models [64]. As undifferentiated cells, SH-SY5Y cells have a neuroblast-like morphology and can present immature neuronal markers. Moreover, SH-SY5Y cells can be differentiated into neuron-like cells through treatment with RA or the brain-derived neurotrophic factor. The differentiated cells present a morphology like that of primary neurons and neuron-specific markers, allowing for its neuronal properties and functions to be studied as well as its impaired metabolism and disease-specific pathologies to be explored [65]. Nonetheless, there are limitations to using SH-SY5Y cells such as viability, metabolic properties, and genomic stability because their cancer-like properties are derived from neuroblastoma. Furthermore, these cells may not fully recapitulate the genetic and epigenetic landscape of primary neurons, potentially limiting their relevance for certain studies [66].

In this current study, the protective mechanisms of ACEE on B[a]P-induced impairment of neurodifferentiation was investigated. First, our results show that ACEE at a concentration of 25 µg/mL prominently reduced the occurrence of B[a]P-inhibited neurite outgrowth in SH-SY5Y cells, suggesting that there may be potential clinical relevance for this concentration of ACEE as an effective dose. Nevertheless, further dose optimization by in vivo and human studies is indispensable. Second, ACEE modified the transcriptome profiles in SH-SY5Y cells. The RNA-seq analysis revealed that DEGs identified in cells co-treated with B[a]P and ACEE were associated with the activation states of reelin signaling in neurons, ephrin receptor signaling, the semaphorin neuronal repulsive signaling pathway, and glutamate receptor signaling. Furthermore, co-treatment of B[a]P and ACEE was associated with other canonical pathways, including the inhibition states of netrin signaling, neuropathic pain signaling in dorsal horn neurons, oxytocin in the brain signaling pathway, synaptic long-term potentiation, and synaptic long-term depression.

Furthermore, the expressions of the *CXCR4*, *ENPP2*, *GAP43*, *GFRA2*, *NELL2*, *NSG2*, *NGB*, *BASP1*, and *NEUROD1* genes were significantly decreased (while *NFASC* was slightly decreased) in the B[a]P-treated SH-SY5Y cells compared to the control (RA). Meanwhile, ACEE reversed the gene expression change induced by B[a]P. ENPP2 is an enzyme associated with neurological, psychiatric, neoplastic, and neurodevelopmental processes [67]. An alteration of neurogenesis and synaptic connectivity, as well as autistic-like features, were found in GAP43-deficient mice [68,69,70]. ENPP2 might be a new therapeutic target for ACEE to relieve NDDs. Reasonably, a previous study indicated that ENPP2 can promote neuronal plasticity through an NMDAR/BDNF/TrkB-dependent mechanism in NDD models [71]. Moreover, the increased expressions of the *NELL2*, *NSG2*, *NGB*, *BASP1*, and *NEUROD1* genes were associated with neurodifferentiation, neuronal polarization, synapse formation, and axon regeneration [40,42,45,48,72]. NGB is a mammalian neuro-specific protein prominently expressed in nerve cells and up-regulated in promoting neurogenesis. NGB can protect neurons from damage and injury [73] through the promotion of neuronal homeostasis and survival [74]. Our Western blot analysis showed that B[a]P treatment down-regulated the protein expressions of GAP43 and NGB, which ACEE could reverse. Interestingly, a study indicated that the natural compounds exerted a protective role against NDDs by modulating NGB expression [75]. Therefore, ACEE could be considered a potential compound to be used for NDDs therapy. Likewise, therapeutic applications in NDDs may also be achieved by modifying these potential signaling molecules using other natural compound-based therapies.

Our transcriptome and Western blot analysis also suggested that two receptors, CXCR4 and GFRA2, may play a role in the protective effect of ACEE against the B[a]P-induced impairment of SH-SY5Y cell differentiation. CXCR4 and GFRA2 are receptors that promote neurodifferentiation, neuronal regeneration, and neuronal proliferation. Significantly increased CXCR4 and GFRA2 expressions were found when the neuroprogenitor cells differentiated into neurons [36,41]. These receptors control neurite outgrowth through multiple intracellular pathways, including the phosphatidylinositol-3-kinase (PI3K)/Akt and ERK-dependent signaling pathways [37,41,76]. According to previous studies, the activation of ERK signaling played an important role in promoting neuronal differentiation [77,78,79]. However, our current study indicated that B[a]P decreased Akt phosphorylation but increased ERK phosphorylation in SH-SY5Y cells, suggesting the inactivation of Akt and activation of ERK-dependent pathways. It has been reported that B[a]P can stimulate the ERK signaling pathway, which causes an increased cell-mediated response, including cancer cell proliferation, migration, and invasion [80,81]. The reason why the B[a]P-induced activation of the ERK-dependent pathway led to decreased neuronal differentiation in our study is not known, but it may be due to the activation of certain yet-to-be-identified mechanisms downstream from ERK, which deserves further investigation. Connecting the Akt/ERK signaling to its downstream effectors/targets would contribute to a better understanding of the toxic mechanisms of B[a]P and also their potential involvement in the progression of B[a]P-induced neurodegeneration.

The binding of phytochemicals in ACEE to CXCR4, GFRA, and RXR was investigated by a docking analysis. CXCR4 is a receptor that is highly expressed in human neural precursor cells located in the cerebellum, hippocampus, and neocortex [82,83]. The interaction between CXCR4 and its ligand, named stroma cell-derived factor 1 (SDF-1), plays an important role in neuronal development and patterning of the cerebellum and hippocampus in the rodent brain [84]. The activation of CXCR4 can facilitate the differentiation of human embryonic stem cell-derived neuronal stem cells [36]. Moreover, CXCR4/SDF-1 signaling promotes neuroplasticity in the injured brain by mediating the proliferation, differentiation, and migration of neuronal precursor cells and also driving the elongation and branching of axons [37]. GFRA, also known as glial cell line-derived neurotrophic factor (GDNF) receptor, plays a vital role in the development and maintenance of the nervous system. The loss of GFRA and GDNF is associated with NDDs, including Parkinson’s disease [85]. GDNF signaling via GFRA1 can stimulate proliferation, differentiation, and axonal growth in cortical GABAergic neurons [86]. Additionally, the complex of GFRA2 and its ligand, neurturin, is considered one of the potential therapeutics for NDDs [31]. In this study, SH-SY5Y cells underwent differentiation using RA treatment. RA can regulate cell growth, survival, death, and neurodifferentiation into functional neurons through retinoic acid receptors, which function as heterodimers with RXR [87]. Given these reasons, three receptors, CXCR4, GFRA, and RXR, were chosen for docking analysis to identify the constituents in ACEE that may act as agonists for these receptors. The results suggested that clionasterol and lupenone may be the potential candidates based on their binding affinity, as reflected in the lower binding energy compared to the standard agonists of each respective target. Nevertheless, docking studies often use simplified models of proteins and ligands, which may not fully capture the complexity of biological systems. These studies typically assume a static conformation of the target protein, ignoring the flexibility or the dynamic nature of proteins in a cellular environment. Further experiments using these potential compounds, including in cell-based assays, should be employed to validate their biological activity and potential therapeutic effects against B[a]P-induced neurotoxicity.

## 5. Conclusions

ACEE denotes neuroprotection from B[a]P-impaired neuronal differentiation, including the induction of GAP43/neuroglobin-mediated neurite outgrowth through the Akt and ERK signaling pathways. Clionasterol and lupenone in ACEE may be the prominent agonists for receptors CXCR4, GFRA, and RXR, which are important in neurodifferentiation. Moreover, ACEE may provide neuroprotective benefits by improving the expression of genes related to neurological diseases and the development and function of the nervous system. Overall, ACEE has the potential as an alternative medicine for neuroprotection. Additionally, the present study identifies new molecular signatures specific to NDD in the context of B[a]P-induced impairment of SH-SY5Y neurodifferentiation, suggesting that this model system could be a valuable tool for screening and evaluating novel anti-NDD agents.

## Figures and Tables

**Table 1:** Lists of primers used for real-time PCR analysis. All primer sequences are shown in 5′ to 3′ orientation.

Gene	Forward Primer	Reverse Primer
*ACTB*	GGCATCCTCACCCTGAAGTA	AGCCTGGATAGCAACGTACA
*GAP43*	AGCCTAAACAAGCCGATGTG	TCAGGCATGTTCTTGGTCAG
*ENPP2*	CACTTTTCATCTGCGAGGGC	CAGCTTTCACCCCTTGCTTG
*CXCR4*	TGGAGGGGATCAATATACACTTC	AGCGTGATGACAAAGAGGAGG
*NGB*	CCAGTTCTCCAGCCCAGAG	CTTCACACCCACTGCCCG
*NELL2*	CAACATTTTGGCTAGGACAGAGA	CTGAGCAATAAATCCCTGGGG
*NEUROD1*	AGAGGTCTGGAATGTGGCAG	GCGTAGGGATGGCTTATGGA
*NSG2*	GTGAAGCTGAACAGTAACCCC	TCTGTTCCGGCTGATATTCC
*GFRA2*	CCTTGGAGGTCTTGCAGGAG	GAGGTCACCGGCTCATAGG
*BASP1*	ATCTTGGGGAAGGAGAAGGC	CCATGGGGTTGCTCTGTCTA
*NFASC*	CTTCAACATCGCCAAGGACC	GTTTTCCTTGGGCCACAGAG

**Table 2:** Overview of the DEG number between different treatment groups in the study.

Comparisons	Up-Regulated DEGs	Down-Regulated DEGs	Total DEGs
RA vs. RA + B[a]P	2182	2007	4189
RA + B[a]P vs. RA + B[a]P + ACEE	1918	2195	4113

**Table 3:** Gene ontology analysis of DEGs among the different treatment conditions in differentiated SH-SY5Y cells. The top 5 canonical pathways, diseases/disorders, and biological functions that are significantly associated with DEGs were predicted using IPA software. Fisher’s exact test was used to calculate the *p* value, which is considered significant at *p* < 0.05. A range of *p* values is provided for the subcategories of diseases/disorders and biological functions under a general category of the database.

Categories	*p* Value	No. of Genes
**RA vs. RA + B[a]P**		
***Canonical Pathways***		
EIF2 signaling	3.31 × 10^−34^	
mTOR signaling	2.32 × 10^−16^	
Regulation of eIF4 and p70S6K signaling	6.57 × 10^−15^	
Unfolded protein response	9.93 × 10^−15^	
Kinetochore metaphase signaling pathway	3.54 × 10^−14^	
***Diseases/Disorders***		
Cancer	8.21 × 10^−17^–5.25 × 10^−277^	4022
Organismal injury and abnormalities	9.07 × 10^−17^–5.25 × 10^−277^	4064
Endocrine system disorders	1.27 × 10^−18^–2.51 × 10^−198^	3502
Gastrointestinal disease	4.48 × 10^−17^–2.56 × 10^−175^	3624
Neurological disease	3.40 × 10^−18^–8.18 × 10^−106^	3014
***Biological Functions***		
Organismal survival	6.09 × 10^−18^–8.13 × 10^−80^	1220
Nervous system development and function	5.33 × 10^−17^–1.04 × 10^−40^	868
Organismal development	9.52 × 10^−17^–1.04 × 10^−40^	1313
Tissue development	8.50 × 10^−17^–1.04 × 10^−40^	760
Tissue morphology	3.89 × 10^−17^–2.94 × 10^−25^	882
**RA + B[a]P vs. RA + B[a]P + ACEE**		
***Canonical Pathways***		
EIF2 signaling	1.89 × 10^−28^	
Coronavirus pathogenesis pathway	4.95 × 10^−13^	
Insulin secretion signaling pathway	2.34 × 10^−12^	
Regulation of eIF4 and p70S6K signaling	4.06 × 10^−12^	
mTOR signaling	8.47 × 10^−12^	
***Diseases/Disorders***		
Cancer	1.09 × 10^−17^–6.11 × 10^−283^	3971
Organismal injury and abnormalities	3.84 × 10^−17^–6.11 × 10^−283^	4011
Endocrine system disorders	2.82 × 10^−19^–2.00 × 10^−207^	3496
Gastrointestinal disease	5.17 x10^−21^–1.42 × 10^−189^	3596
Neurological disease	3.84 × 10^−17^–2.26 × 10^−116^	2969
***Biological Functions***		
Organismal survival	2.10 × 10^−17^–1.30 × 10^−71^	1181
Nervous system development and function	2.76 × 10^−17^–1.44 × 10^−54^	904
Organismal development	2.76 × 10^−17^–1.44 × 10^−54^	1582
Tissue development	8.08 × 10^−18^–1.44 × 10^−54^	1146
Embryonic development	1.42 × 10^−17^–7.73 × 10^−36^	1046

**Table 4:** DEG-associated neurological diseases and nervous system development/functions among the different treatment conditions in differentiated SH-SY5Y cells. The top neurological diseases and nervous system development/functions that are significantly associated with DEGs were predicted using IPA software. *p* values were calculated by Fisher’s exact test, which is considered significant at *p* < 0.05.

Name	*p* Value	No. of Genes
**RA vs. RA + B[a]P**		
***Neurological Disease***		
Brain tumor	8.63 × 10^−95^	2379
Motor dysfunction or movement disorder	6.52 × 10^−48^	637
Neuromuscular disease	7.54 × 10^−34^	526
Cognitive impairment	1.83 × 10^−33^	446
Progressive neurological disorder	6.37 × 10^−21^	485
***Nervous System Development and Function***		
Neuritogenesis	2.04 × 10^−40^	407
Development of neurons	9.13 × 10^−37^	480
Morphology of nervous system	1.2 × 10^−33^	478
Proliferation of neuronal cells	3.56 × 10^−30^	276
Dendritic growth/branching	8.16 × 10^−20^	170
**RA + B[a]P vs. RA + B[a]P + ACEE**		
***Neurological Disease***		
Brain tumor	1.6 × 10^−99^	2364
Cognitive impairment	8.76 × 10^−38^	454
Neuromuscular disease	1.82 × 10^−27^	498
Dementia	2.57 × 10^−17^	287
Tauopathy	3.63 × 10^−17^	273
***Nervous System Development and Function***		
Neuritogenesis	1.44 × 10^−54^	437
Development of neurons	4.84 × 10^−54^	522
Proliferation of neuronal cells	1.14 × 10^−37^	291
Morphology of nervous system	1.26 × 10^−37^	485
Dendritic growth/branching	4.82 × 10^−23^	176

**Table 5:** List of selected DEGs and their reported changes in neuronal differentiation.

Genes		Changes Observed in Neuronal Differentiation
*CXCR4*	C-X-C motif chemokine receptor 4	Up-regulation in neuronal differentiation [36] Up-regulation in neuronal regeneration following cerebral ischemia [37]
*ENPP2*	Ectonucleotide pyrophosphatase/phosphodiesterase 2	Up-regulation in oligodendrocyte differentiation in the zebrafish hindbrain [38]
*GAP43*	Growth-associated protein 43	Up-regulation in neuronal differentiation [39] Up-regulation in axon regeneration for the treatment of NDs [40]
*GFRA2*	GDNF family receptor alpha 2	Up-regulation in the promotion of neuronal proliferation [41]
*NELL2*	Neural EGFL-like 2	Up-regulation in neuronal differentiation, polarization, and axon guidance [42,43]
*NFASC*	Neurofascin	Up-regulation in nervous system development and function of node of Ranvier [44]
*NSG2*	Neuronal vesicle trafficking associated 2	Up-regulation required in normal synapse formation and/or maintenance [45]
*NGB*	Neuroglobin	Up-regulation in the promotion of neurogenesis [46,47]
*BASP1*	Brain-abundant membrane-attached signal protein 1	Up-regulation in axon regeneration for the treatment of NDDs [40]
*NEUROD1*	Neuronal differentiation 1	Up-regulation in neuronal differentiation and brain development [48,49]

**Table 6:** Lists of three best-ranked compounds identified as ligands on selected target proteins.

No.	CXCR4	GFRA1	GFRA2	RXRα	RXRβ	RXRγ
1	Clionasterol (−9.20)	Lupenone (−7.66)	Lupenone (−6.86)	Clionasterol (−11.75)	β-amyrone (−12.08)	Clionasterol (−9.13)
2	β-amyrin (−8.69)	Friedelan-3-one (−5.75)	Friedelan-3-one (−6.52)	β-amyrone (−10.87)	Clionasterol (−11.39)	Lupenone (−8.12)
3	Lupenone (−8.69)	Clionasterol (−5.43)	Clionasterol (−6.15)	Lupenone (−9.37)	Lupenone (−11.33)	β-amyrone (−6.79)
Std *	NUCC-390 (−8.11)	BT13 (−5.31)	BT13 (−5.55)	9-cis-retinoic acid (−11.46)	9-cis-retinoic acid (−10.63)	9-cis-retinoic acid (−7.79)

* Standard positive control docked in each protein.

## Data Availability

The datasets generated and/or analyzed during this study are available in the Gene Expression Omnibus (GEO) repository, accession number GSE263834.

## Supplementary Materials

The following supporting information can be downloaded at https://www.mdpi.com/article/10.3390/nu16162727/s1, Table S1: The list of genes which were differentially expressed in RA + B[a]P treatment compared with control cells; Table S2: The list of genes which were differentially expressed in RA + B[a]P + ACEE compared with RA + B[a]P treatment cells; Table S3: Molecular docking study between ACEE phytochemicals and the binding site of C-X-C motif chemokine receptor 4 (CXCR4); Table S4: Molecular docking study between ACEE phytochemicals and the binding site of GDNF family receptor alpha 1 (GFRA1); Table S5: Molecular docking study between ACEE phytochemicals and the binding site of GDNF family receptor alpha 2 (GFRA2); Table S6: Molecular docking study between ACEE phytochemicals and the binding site of retinoid X receptor alpha (RXRα); Table S7: Molecular docking study between ACEE phytochemicals and the binding site of retinoid X receptor beta (RXRβ); Table S8: Molecular docking study between ACEE phytochemicals and the binding site of retinoid X receptor gamma (RXRγ).
