# Supplementary material for: Aquilaria crassna Extract Exerts Neuroprotective Effect against Benzo[a]pyrene-Induced Toxicity in Human SH-SY5Y Cells: An RNA-Seq-Based Transcriptome Analysis
Source: Nutrients. 2024 Aug 16;16(16):2727. doi: 10.3390/nu16162727 (PMC11357018; doi:10.3390/nu16162727)
Supplement: Supplementary file 1 [file nutrients-16-02727-s001.zip › Supplementary Tables S3-S8 (Docking results)edit.pdf]

**Supplementary Table S3.** Molecular docking study between ACEE phytochemicals and the binding site of C-X-C motif chemokine receptor 4 (CXCR4)

CXCR4 (PDB: 3ODU)

**Parameters**

Grid size (x × z × y): 40 × 40 × 40

Spacing: 0.375 Å

Grid center (x, y, z): 20.39, -8.747, 71.653

| No. | Compound | Binding energy (kcal/mol) | Amino acid interaction | |
|---|---|---|---|---|
| | | | Hydrogen bond | Hydrophobic bond |
| | IT1t (redocked original ligand) | -7.84 | TYR45 | TYR45<br>TRP94<br>TRP94<br>TRP94<br>TRP94<br>ALA98<br>TRP102<br>VAL112<br>HIS113<br>ILE185<br>CYS186 |
| | NUCC-390 (agonist) | -8.11 | TYR45<br>HIS113<br>GLU288 | LEU41<br>TYR45<br>TRP94<br>TRP94<br>TRP94<br>ALA98<br>ALA98<br>TRP102<br>HIS113<br>CYS186 |
| 1 | Neophytadiene | -5.52 | | TYR45<br>TRP94<br>TRP94<br>TRP94<br>TRP94<br>TRP94<br>TRP94<br>TRP94<br>TRP94<br>TRP94<br>TRP102<br>VAL112<br>VAL112<br>HIS113<br>HIS113<br>HIS113 |

| | | | | |
|---|---|---|---|---|
| | | | | HIS113<br>TYR116<br>TYR116<br>TYR116<br>CYS186 |
| 2 | Vitamin E | -7.92 | HIS281<br>GLU32 | TYR116<br>TRP94<br>TRP94<br>LEU41<br>VAL112<br>VAL112<br>TRP94<br>TRP94<br>TRP94<br>TRP94<br>TRP94<br>TRP102<br>HIS113<br>TYR116<br>HIS281 |
| 3 | beta-Amyrone | -8.07 | | TRP94<br>TRP94<br>TRP94<br>TRP94<br>TRP94<br>TRP94<br>TRP94<br>TRP94<br>TRP102<br>VAL112<br>HIS113<br>HIS113<br>HIS113<br>TYR116<br>TYR116<br>TYR116 |
| 4 | beta-Amyrin | -8.69 | ASN33 | LEU41<br><br>TRP94<br>TRP94 |

| | | | | |
|---|---|---|---|---|
| | | | | TRP94<br>TRP94<br>ALA98<br>HIS113<br>HIS113<br>TYR116 |
| 5 | Friedelan-3-one | -7.17 | | TRP94<br>TRP94<br>TRP94<br>TRP102<br>VAL112<br>VAL112<br>HIS113<br>HIS113<br>HIS113<br>CYS186 |
| 6 | Clionasterol (gamma-Sitosterol) | -9.20 | PHE93 | TRP94<br>TRP94<br>TRP94<br>TRP94<br>TRP94<br>HIS113<br>HIS113<br>HIS113<br>TYR116<br>TYR116 |
| 7 | Squalene | -6.46 | | LEU41<br>TRP94<br>TRP94<br>TRP94<br>ALA98<br>TRP102<br>VAL112<br>HIS113<br>HIS113<br>TYR116<br>TYR116<br>ILE185 |
| 8 | 3,7,11,15-Tetramethyl-2-hexadecen-1-ol | -5.06 | GLU32 | LEU41<br>TRP94 |

| | | | | |
|---|---|---|---|---|
| | | | | TRP94<br>TRP94<br>TRP94<br>TRP94<br>TRP94<br>ALA98<br>ALA98<br>TRP102<br>HIS113<br>HIS113<br>TYR116<br>TYR116 |
| 9 | n-Hexadecanoic acid (Palmitic acid) | -3.97 | ARG188<br>ARG188 | TYR45<br>TRP94<br>TRP94<br>TRP94<br>TRP94<br>TRP94<br>TRP94<br>TRP94<br>ALA98<br>TRP102<br>VAL112<br>VAL112<br>HIS113<br>HIS113<br>TYR116<br>CYS186<br>CYS186 |
| 10 | 9-Octadecenamide, (Z)- | -5.06 | TYR45<br>TRP94<br>ASP97 | TRP94<br>TRP94<br>TRP94<br>TRP94<br>TRP94<br>TRP102<br>VAL112<br>VAL112<br>HIS113<br>HIS113<br>HIS113 |

| | | | | |
|---|---|---|---|---|
| | | | | TYR116<br>CYS186<br>CYS186 |
| 11 | Lupenone | -8.69 | ARG183<br>ILE185<br>CYS186 | TRP94<br>TRP94<br>TRP94<br>TRP94<br>TRP94<br>TRP94<br>TRP94<br>TRP102<br>VAL112<br>HIS113<br>HIS113<br>HIS113<br>TYR116<br>TYR116 |

**Supplementary Table S4.** Molecular docking study between ACEE phytochemicals and the binding site of GDNF family receptor alpha 1 (GFRA1)

GFRA1 (4UX8)

**Parameters**

Grid size (x × z × y): 60 × 60 × 60

Spacing: 0.375 Å

Grid center (x, y, z): -8.924, -36.393, -3.105

| No. | Compound | Binding energy (kcal/mol) | Amino acid interaction | |
|---|---|---|---|---|
| | | | Hydrogen bond | Hydrophobic bond |
| | Aminoquinol (XIB4035) (agonist) | -6.27 | GLN227 | VAL230<br>**ARG171**<br>**MET211**<br>**VAL230**<br>**VAL232**<br>TYR174<br>ARG224 |
| | BT13 (agonist) | -5.31 | ARG171<br>SER172 | LYS168<br>ARG171<br>ILE175<br>ILE175<br>ILE175<br>**MET211**<br>**ARG224**<br>ARG224 |
| 1 | Neophytadiene | -4.65 | | **ARG171**<br>TYR174<br>**ILE175**<br>**ILE175**<br>**ILE175**<br>ILE175<br>**MET211**<br>**MET211**<br>**ARG224**<br>VAL230 |
| 2 | Vitamin E | -4.99 | | **CYS154**<br>ILE175<br>**ILE175**<br>**ILE175**<br>**ILE175**<br>**MET211**<br>**ARG224**<br>VAL230 |
| 3 | beta-Amyrone | -5.41 | | **ILE175**<br>**MET211**<br>**MET211**<br>ARG224 |

| | | | | |
|---|---|---|---|---|
| | | | | ARG224<br>VAL230 |
| 4 | beta-Amyrin | -4.74 | ASN162 | **ARG171**<br>ILE175<br>ILE175<br>ARG224 |
| 5 | Friedelan-3-one | -5.75 | ARG171 | VAL230 |
| 6 | Clionasterol (gamma-Sitosterol) | -5.43 | THR228 | ILE175<br>ARG224 |
| 7 | Squalene | -5.05 | | **CYS154**<br>ALA158<br>**LYS168**<br>**ARG171**<br>**ARG171**<br>**ILE175**<br>**ILE175**<br>**ILE175**<br>**MET211**<br>**CYS214**<br>**ARG224**<br>ARG224<br>ARG224<br>VAL230 |
| 8 | 3,7,11,15-Tetramethyl-2-hexadecen-1-ol | -3.46 | MET211<br>CYS214 | ALA158<br>**ILE175**<br>**ILE175**<br>**ILE175**<br>**MET211**<br>ARG224<br>**ARG224**<br>**VAL230**<br>VAL230 |
| 9 | n-Hexadecanoic acid (Palmitic acid) | -4.21 | ARG171<br>ARG224<br>ARG224 | **CYS154**<br>**CYS214**<br>ARG224 |
| 10 | 9-Octadecenamide, (Z)- | -4.46 | CYS154<br>MET211<br>ARG224 | ILE175<br>ARG224<br>VAL230 |
| 11 | Lupenone | -7.66 | ARG171<br>ARG171 | **CYS154**<br>**CYS154** |

| | | | | |
|---|---|---|---|---|
| | | | ARG224 | ALA157<br>ALA158<br>**ARG171**<br>ILE175<br>ILE175<br>**ILE175**<br>**ILE175**<br>**ILE175**<br>**MET211**<br>**ARG224**<br>**ARG224**<br>**ARG225**<br>VAL230 |

**Supplementary Table S5.** Molecular docking study between ACEE phytochemicals and the binding site of GDNF family receptor alpha 2 (GFRA2)

GFRA2 (5MR4)

**Parameters**

Grid size (x × z × y): 60 × 60 × 60

Spacing: 0.375 Å

Grid center (x, y, z): 82.786, -68.61, -41.835

| No. | Compound | Binding energy (kcal/mol) | Amino acid interaction | |
|---|---|---|---|---|
| | | | **Hydrogen bond** | **Hydrophobic bond** |
| | Aminoquinol (XIB4035)<br>(GFRA1 agonist) | -5.23 | ARG178<br>**ARG232** | ALA228<br>ARG232<br>THR236<br>**LEU238**<br>LEU238 |
| | BT13 (GFRA1 agonist) | -5.55 | ASN169<br>ASN169<br>ARG178<br>ARG178<br>SER179<br>THR236 | LYS175<br>**ARG178**<br>TYR181<br>ILE182<br>ILE182<br>**ILE182**<br>**ARG232**<br>LEU238 |
| 1 | Neophytadiene | -3.47 | | **ILE182**<br>**ILE182**<br>**ILE182**<br>**MET219**<br>ARG232<br>ARG232<br>**LEU238**<br>LEU238 |
| 2 | Vitamin E | -5.44 | ARG178<br>TYR181<br>THR236 | **LYS175**<br>TYR181<br>**ILE182**<br>**ILE182**<br>ILE182<br>**MET219**<br>ARG232<br>**ARG232**<br>**LEU238**<br>LEU238 |
| 3 | beta-Amyrone | -6.06 | | ALA165<br>**ILE182**<br>**MET219**<br>**MET219**<br>**ARG232**<br>ARG232 |

| | | | | |
|---|---|---|---|---|
| | | | | **LEU238**<br>LEU238 |
| 4 | beta-Amyrin | -6.01 | SER183 | ALA165<br>ILE182<br>ILE182<br>ILE182<br>**ILE182**<br>ARG232<br>ARG232<br>LEU238 |
| 5 | Friedelan-3-one | -6.52 | ARG178 | TYR181<br>**ILE182**<br>ILE182 |
| 6 | Clionasterol (gamma-Sitosterol) | -6.15 | | ILE182<br>ILE182<br>**MET219**<br>**ARG232**<br>ARG232 |
| 7 | Squalene | -4.63 | | TYR181<br>**ILE182**<br>**ILE182**<br>**ILE182**<br>**ILE182**<br>**ILE182**<br>**ILE182**<br>**ILE182**<br>**MET219**<br>ARG232<br>ARG232<br>**ARG232**<br>LEU238 |
| 8 | 3,7,11,15-Tetramethyl-2-hexadecen-1-ol | -4.19 | ARG232 | **ILE182**<br>**ILE182**<br>**MET219**<br>ARG232<br>LEU238 |
| 9 | n-Hexadecanoic acid (Palmitic acid) | -5.10 | ARG178<br>**ARG178**<br>ARG232 | TYR181<br>**ILE182**<br>**ILE182**<br>**MET219** |

| | | | | |
|---|---|---|---|---|
| | | | | **ARG232**<br>**LEU238**<br>LEU238 |
| 10 | 9-Octadecenamide, (Z)- | -3.98 | ARG178<br>ARG178<br>ARG232 | TYR181<br>**ILE182**<br>**ILE182**<br>**ILE182**<br>ARG232<br>ARG232<br>LEU238 |
| 11 | Lupenone | -6.86 | **ARG178**<br>ARG178 | ALA165<br>**ARG178**<br>ILE182<br>ILE182<br>**ILE182**<br>**ILE182**<br>**MET219**<br>**MET219**<br>ARG232 |

**Supplementary Table S6:** Molecular docking study between ACEE phytochemicals and the binding site of Retinoid X receptor alpha (RXRα)

RXRα (PDB:1FM9)

**Parameters**

Grid size (x × z × y): 40 × 40 × 40

Spacing: 0.375 Å

Grid center (x, y, z): 14.706, -9.397, 44.439

| No. | Compound | Binding energy (kcal/mol) | Amino acid interaction | |
|---|---|---|---|---|
| | | | Hydrogen bond | Hydrophobic bond |
| | 9-cis-retinoic acid (redocked original ligand) | -11.46 | ARG316 | VAL265 |
| | | | ARG316 | VAL265 |
| | | | ALA327 | ILE268 |
| | | | | ILE268 |
| | | | | ILE268 |
| | | | | ILE268 |
| | | | | ILE268 |
| | | | | CYS269 |
| | | | | ALA271 |
| | | | | ALA272 |
| | | | | ALA272 |
| | | | | TRP305 |
| | | | | ILE310 |
| | | | | PHE313 |
| | | | | PHE313 |
| | | | | LEU326 |
| | | | | VAL342 |
| | | | | CYS432 |
| | | | | CYS432 |
| | | | | LEU436 |
| | | | | LEU436 |
| 1 | Neophytadiene | -7.70 | | VAL265 |
| | | | | ILE268 |
| | | | | ILE268 |
| | | | | ILE268 |
| | | | | CYS269 |
| | | | | CYS269 |
| | | | | ALA271 |
| | | | | ALA271 |
| | | | | ALA272 |
| | | | | ALA272 |
| | | | | ALA272 |
| | | | | TRP305 |
| | | | | LEU309 |
| | | | | LEU309 |
| | | | | LEU309 |
| | | | | PHE313 |

| | | | | |
|---|---|---|---|---|
| | | | | PHE313<br>PHE313<br>LEU326<br>ALA327<br>CYS432<br>LEU436<br>LEU436<br>LEU436 |
| 2 | Vitamin E | -9.92 | | ILE268<br>ILE268<br>ILE268<br>ILE268<br>ILE268<br>ALA271<br>ALA272<br>ALA272<br>ALA272<br>LEU309<br>LEU309<br>ILE310<br>ILE310<br>PHE313<br>PHE313<br>PHE313<br>PHE313<br>PHE313<br>ILE324<br>ILE324<br>LEU326<br>VAL342<br>VAL342<br>ILE345<br>ILE345<br>PHE346<br>VAL349<br>CYS432<br>CYS432<br>HIS435<br>LEU436<br>LEU436 |

| | | | | |
|---|---|---|---|---|
| 3 | beta-Amyrone | -10.87 | | ILE268<br>ALA271<br>ALA271<br>TRP305<br>LEU309<br>ILE310<br>PHE313<br>PHE313<br>PHE313<br>PHE313<br>ILE324<br>LEU326<br>LEU326<br>LEU326<br>ILE324<br>ILE345<br>CYS432<br>CYS432<br>HIS435<br>LEU436 |
| 4 | beta-Amyrin | -1.38 | ARG316 | ILE268<br>ILE268<br>ILE268<br>ALA271<br>ALA271<br>ALA272<br>ALA272<br>ALA272<br>LEU309<br>LEU309<br>LEU309<br>LEU309<br>ILE310<br>PHE313<br>PHE313<br>PHE313<br>PHE313<br>LEU326<br>VAL342<br>ILE345 |

| | | | | |
|---|---|---|---|---|
| | | | | ILE345<br>ILE345<br>PHE346<br>VAL349<br>CYS432<br>CYS432<br>CYS432<br>CYS432<br>LEU436 |
| 5 | Friedelan-3-one | -6.97 | | VAL265<br>VAL265<br>ILE268<br>CYS269<br>VAL342<br>CYS432<br>HIS435<br>LEU436 |
| 6 | Clionasterol (gamma-Sitosterol) | -11.75 | VAL342 | ILE268<br>ILE268<br>ALA271<br>ALA272<br>ALA272<br>TRP305<br>LEU309<br>LEU309<br>ILE310<br>PHE313<br>LEU326<br>VAL342<br>ILE345<br>ILE345<br>CYS432<br>CYS432<br>LEU436 |
| 7 | Squalene | -10.05 | | VAL265<br>ILE268<br>ILE268<br>ILE268<br>ILE268<br>ILE268 |

| | | | | |
|---|---|---|---|---|
| | | | | ILE268<br>ALA271<br>ALA272<br>TRP305<br>TRP305<br>PHE313<br>PHE313<br>PHE313<br>PHE313<br>ILE324<br>ILE324<br>LEU326<br>LEU326<br>LEU326<br>VAL342<br>VAL342<br>ILE345<br>PHE346<br>PHE346<br>VAL349<br>VAL349<br>CYS432<br>CYS432<br>LEU436<br>CYS432<br>LEU436<br>LEU436<br>PHE439<br>LEU451 |
| 8 | 3,7,11,15-Tetramethyl-2-hexadecen-1-ol | -7.10 | ALA327 | VAL265<br>ILE268<br>ALA271<br>ALA272<br>ALA272<br>LEU309<br>LEU309<br>ILE310<br>PHE313<br>PHE313<br>VAL342 |

| | | | | |
|---|---|---|---|---|
| | | | | ILE345<br>CYS432<br>CYS432<br>CYS432<br>CYS432<br>HIS435<br>HIS435<br>LEU436<br>PHE439 |
| 9 | n-Hexadecanoic acid (Palmitic acid) | -6.73 | ARG316<br>ALA327 | VAL265<br>ILE268<br>ALA271<br>ALA272<br>ALA272<br>ALA272<br>TRP305<br>LEU309<br>LEU309<br>ILE310<br>PHE313<br>CYS432<br>LEU436 |
| 10 | 9-Octadecenamide, (Z)- | -6.37 | ARG316 | ILE268<br>ILE268<br>ALA271<br>ALA272<br>ALA272<br>TRP305<br>LEU309<br>LEU309<br>ILE310<br>ILE310<br>ILE310<br>PHE313<br>PHE313<br>PHE313<br>PHE313<br>ILE324<br>LEU326<br>VAL349 |

| | | | | |
|---|---|---|---|---|
| | | | | CYS432<br>CYS432 |
| 11 | Lupenone | -9.37 | | VAL265<br>ILE268<br>ALA271<br>ALA272<br>ALA327<br>VAL342<br>VAL342<br>VAL342<br>ILE345<br>ILE345<br>ILE345<br>PHE346<br>VAL349<br>CYS432<br>CYS432<br>CYS432<br>CYS432<br>HIS435<br>HIS435<br>LEU436<br>LEU436<br>LEU436<br>PHE439<br>PHE439 |

**Supplementary Table S7:** Molecular docking study between ACEE phytochemicals and the binding site of Retinoid X receptor beta (RXRβ)

RXRβ (PDB:1UHL)

**Parameters**

Grid size (x × z × y): 40 × 40 × 40

Spacing: 0.375 Å

Grid center (x, y, z): 44.653, -7.883, 30.074

| No. | Compound | Binding energy (kcal/mol) | Amino acid interaction | |
|---|---|---|---|---|
| | | | Hydrogen bond | Hydrophobic bond |
| | Metoprenic acid (redocked original ligand) | -8.10 | LEU397<br>ALA398 | ILE339<br>ILE339<br>ILE339<br>ALA342<br>ALA343<br>ALA343<br>TRP376<br>LEU380<br>ILE381<br>PHE384<br>PHE384<br>PHE384<br>LEU397<br>CYS503<br>LEU507 |
| | 9-cis-retinoic acid (agonist) | -10.63 | ARG387<br>LEU397<br>ALA398 | ILE339<br>ILE339<br>ILE339<br>ALA343<br>LEU380<br>ILE381<br>PHE384<br>PHE384<br>PHE384<br>PHE384<br>VAL413<br>VAL413<br>ILE416<br>CYS503<br>CYS503<br>HIS506<br>LEU507<br>LEU507 |
| 1 | Neophytadiene | -7.44 | | ILE339<br>ILE339<br>ILE339<br>ILE339 |

| | | | | |
|---|---|---|---|---|
| | | | | ALA342<br>ALA343<br>ALA343<br>TRP376<br>LEU380<br>LEU380<br>LEU380<br>ILE381<br>PHE384<br>PHE384<br>PHE384<br>PHE384<br>PHE384<br>PHE384<br>LEU397<br>LEU397<br>PHE417<br>VAL420<br>CYS503 |
| 2 | Vitamin E | -9.77 | ARG387 | VAL336<br>ILE339<br>ILE339<br>ILE339<br>ILE339<br>ILE339<br>ALA342<br>ALA342<br>ALA343<br>ALA343<br>ALA343<br>ALA343<br>LEU380<br>LEU380<br>LEU380<br>ILE381<br>PHE384<br>PHE384<br>PHE384<br>PHE384<br>PHE384 |

| | | | | |
|---|---|---|---|---|
| | | | | LEU397<br>LEU397<br>VAL403<br>ALA408<br>VAL413<br>VAL413<br>VAL413<br>ILE416<br>PHE417<br>VAL420<br>CYS503<br>CYS503<br>LEU507<br>LEU507<br>PHE510 |
| 3 | beta-Amyrone | -12.08 | ARG387<br>LEU397<br>ALA398 | VAL336<br>ILE339<br>ILE339<br>ALA343<br>ALA343<br>LEU380<br>PHE384<br>PHE384<br>ILE395<br>VAL413<br>ILE416<br>PHE417<br>VAL420<br>VAL420<br>CYS503<br>CYS503<br>LEU507<br>LEU507<br>PHE510 |
| 4 | beta-Amyrin | -5.68 | ARG387 | ILE339<br>ILE339<br>ILE339<br>ALA342<br>ALA342<br>ALA343 |

| | | | | |
|---|---|---|---|---|
| | | | | ALA343<br>ALA343<br>LEU380<br>LEU380<br>LEU380<br>LEU380<br>ILE381<br>PHE384<br>PHE384<br>PHE384<br>PHE384<br>VAL413<br>ILE416<br>ILE416<br>ILE416<br>PHE417<br>VAL420<br>CYS503<br>CYS503<br>CYS503<br>LEU507<br>LEU507 |
| 5 | Friedelan-3-one | -6.79 | | ILE339<br>ILE381<br>PHE384<br>VAL413<br>VAL413<br>VAL420<br>CYS503<br>CYS503<br>LEU507 |
| 6 | Clionasterol (gamma-Sitosterol) | -11.39 | ARG387 | ILE339<br>ILE339<br>ALA342<br>ALA342<br>ALA343<br>ALA343<br>ALA343<br>LEU380<br>ILE381 |

| | | | | |
|---|---|---|---|---|
| | | | | PHE384<br>PHE384<br>PHE384<br>LEU397<br>PHE417<br>PHE417<br>VAL420<br>CYS503<br>CYS503<br>HIS506<br>LEU507<br>LEU507 |
| 7 | Squalene | -9.20 | | VAL336<br>ILE339<br>ILE339<br>ILE339<br>ILE339<br>ILE339<br>ILE339<br>ALA342<br>ALA343<br>ALA343<br>ALA343<br>TRP376<br>LEU380<br>ILE381<br>ILE381<br>PHE384<br>PHE384<br>PHE384<br>PHE384<br>ILE395<br>ILE395<br>LEU397<br>LEU397<br>LEU397<br>LEU397<br>VAL403<br>VAL413<br>VAL413 |

| | | | | |
|---|---|---|---|---|
| | | | | VAL413<br>ILE416<br>PHE417<br>PHE417<br>VAL420<br>VAL420<br>ILE499<br>CYS503<br>CYS503<br>CYS503<br>CYS503<br>LEU507<br>LEU507<br>PHE510 |
| 8 | 3,7,11,15-Tetramethyl-2-hexadecen-1-ol | -7.17 | ALA398 | VAL336<br>ILE339<br>ILE339<br>ALA343<br>TRP376<br>LEU380<br>ILE381<br>PHE384<br>PHE384<br>PHE384<br>LEU397<br>VAL413<br>VAL413<br>CYS503<br>CYS503<br>CYS503<br>CYS503<br>LEU507<br>LEU507<br>LEU507<br>PHE510 |
| 9 | n-Hexadecanoic acid (Palmitic acid) | -6.22 | ARG387<br>ALA398 | ILE339<br>ALA343<br>LEU380<br>LEU380<br>ILE381 |

| | | | | |
|---|---|---|---|---|
| | | | | ILE381<br>PHE384<br>PHE384<br>PHE384<br>ILE416<br>PHE417<br>CYS503 |
| 10 | 9-Octadecenamide, (Z)- | -6.77 | GLN346<br>LEU380 | ILE339<br>ILE339<br>ALA342<br>ALA343<br>PHE384<br>PHE384<br>PHE384<br>ILE395<br>ALA398<br>VAL413<br>ILE416<br>PHE417<br>PHE417<br>VAL420<br>CYS503<br>CYS503<br>HIS506 |
| 11 | Lupenone | -11.33 | ARG387 | ILE339<br>ILE339<br>ILE339<br>ALA342<br>ALA343<br>LEU380<br>ILE381<br>PHE384<br>PHE384<br>ILE395<br>ALA398<br>VAL403<br>ALA408<br>VAL413<br>VAL413<br>VAL413 |

| | | | | |
|---|---|---|---|---|
| | | | | ILE416<br>PHE417<br>PHE417<br>CYS503<br>CYS503<br>LEU507<br>LEU507 |

**Supplementary Table S8:** Molecular docking study between ACEE phytochemicals and the binding site of Retinoid X receptor gamma (RXRγ)

RXRγ (PDB:2GL8)

**Parameters**

Grid size (x × z × y): 40 × 40 × 40

Spacing: 0.375 Å

Grid center (x, y, z): 1.78, 14.533, 3.298

| No. | Compound | Binding energy (kcal/mol) | Amino acid interaction | |
|---|---|---|---|---|
| | | | **Hydrogen bond** | **Hydrophobic bond** |
| | 9-cis-retinoic acid (agonist) | -7.79 | ARG95<br>ARG95 | LEU55<br>LEU88<br>LEU88<br>ILE89<br>ILE89<br>PHE92<br>PHE92<br>PHE92<br>LEU105<br>LEU105<br>LEU109<br>CYS211<br>CYS211<br>LEU215<br>LEU215 |
| 1 | Neophytadiene | -5.30 | | LEU55<br>LEU55<br>LEU88<br>ILE89<br>PHE92<br>PHE92<br>PHE92<br>ILE103<br>LEU105<br>LEU105<br>ALA106<br>LEU215<br>LEU215<br>LEU215<br>LEU215 |
| 2 | Vitamin E | -6.32 | | VAL24<br>LEU55<br>LEU55<br>LEU55<br>LEU55<br>TRP84<br>TRP84 |

| | | | | |
|---|---|---|---|---|
| | | | | LEU88<br>LEU88<br>LEU88<br>ILE89<br>PHE92<br>LEU105<br>LEU105<br>LEU105<br>ALA106<br>ALA106<br>LEU215 |
| 3 | beta-Amyrone | -6.79 | | LEU55<br>LEU55<br>LEU55<br>LEU55<br>LEU88<br>LEU88<br>PHE92<br>LEU105<br>ALA106<br>ALA106<br>ALA106 |
| 4 | beta-Amyrin | +6.55 | | LEU55<br>LEU55<br>LEU55<br>ILE89<br>ILE89<br>PHE92<br>LEU105<br>LEU105<br>CYS211<br>CYS211<br>CYS211<br>LEU215<br>LEU215 |
| 5 | Friedelan-3-one | -6.26 | ARG95<br>ARG95<br>ALA106 | LEU55<br>LEU88<br>PHE92 |
| 6 | Clionasterol (gamma-Sitosterol) | -9.13 | ASN85 | LEU55<br>LEU55 |

| | | | | |
|---|---|---|---|---|
| | | | | TRP84<br>LEU88<br>LEU88<br>ILE89<br>PHE92<br>PHE92<br>PHE92<br>LEU105<br>LEU105<br>ALA106 |
| 7 | Squalene | -5.90 | | LEU55<br>LEU55<br>LEU55<br>TRP84<br>LEU88<br>LEU88<br>LEU88<br>LEU88<br>LEU88<br>PHE92<br>PHE92<br>LEU105<br>ALA106<br>ALA106 |
| 8 | 3,7,11,15-Tetramethyl-2-hexadecen-1-ol | -5.30 | LEU88<br>ARG95<br>ARG95 | LEU55<br>LEU55<br>PHE92<br>LEU105<br>LEU105<br>ALA106<br>CYS211<br>LEU215<br>LEU215 |
| 9 | n-Hexadecanoic acid (Palmitic acid) | -5.23 | ARG95 | LEU55<br>TRP84<br>LEU88<br>PHE92<br>LEU105<br>LEU215 |
| 10 | 9-Octadecenamide, (Z)- | -4.59 | ASN85 | LEU55 |

| | | | | |
|---|---|---|---|---|
| | | | | LEU88<br>LEU88<br>PHE92<br>PHE92<br>LEU105<br>LEU105<br>LEU105<br>LEU105<br>ALA106 |
| 11 | Lupenone | -8.12 | | LEU55<br>LEU55<br>LEU88<br>ILE89<br>PHE92<br>PHE92<br>PHE92<br>ILE103<br>LEU105<br>LEU105<br>ALA106<br>LEU215<br>LEU215<br>LEU215<br>LEU215 |
